# Supplementary material for: The Construction and Exploration of a Comprehensive MicroRNA Centered Regulatory Network in Foxtail Millet (Setaria italica L.)
Source: Front Plant Sci. 2022 May 6;13:848474. doi: 10.3389/fpls.2022.848474 (PMC9121102; doi:10.3389/fpls.2022.848474)

T=Seita.5G202700.1_Q=Sit−miRN3001b_S=1731

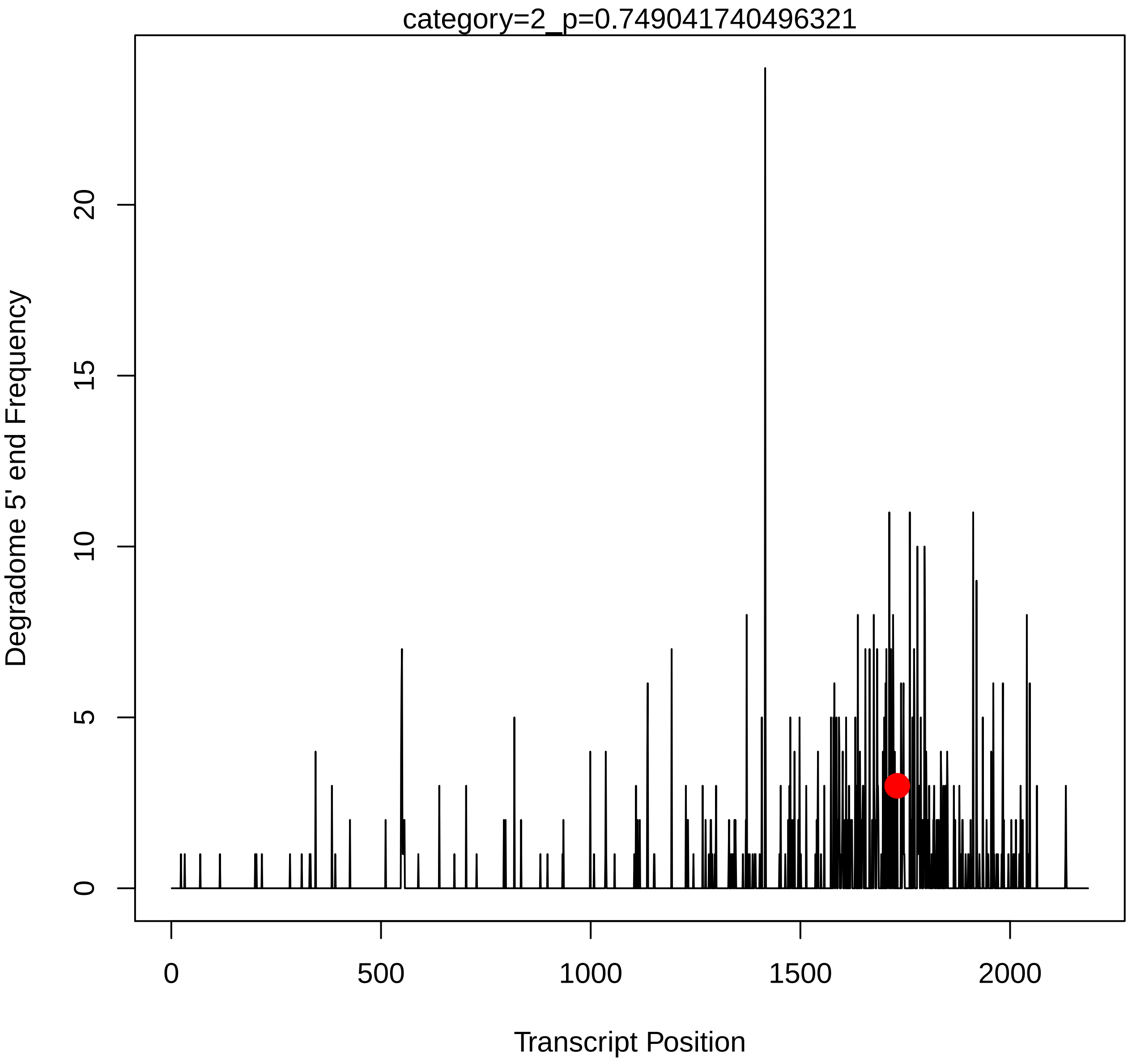

Supplement: Supplementary file 5 [file Data_Sheet_5.zip › Sit-miRN3001b_Seita.5G202700.1_1731_TPlot.pdf]
